# Fibromyalgia, milnacipran and experimental pain modulation: study protocol for a double blind randomized controlled trial

**DOI:** 10.1186/s13063-015-0659-4

**Published:** 2015-04-03

**Authors:** Nicolas Macian, Bruno Pereira, Coralie Shinjo, Claude Dubray, Gisèle Pickering

**Affiliations:** CHU de Clermont-Ferrand, Inserm CIC 1405, Centre de Pharmacologie Clinique, F-63003 Clermont-Ferrand, France; CHU de Clermont-Ferrand, Délégation Recherche Clinique & Innovation - Villa annexe IFSI, 58 Rue Montalembert, F-63003 Clermont-Ferrand, France; Clermont Université, Université d’Auvergne, Pharmacologie Fondamentale et Clinique de la Douleur, Laboratoire de Pharmacologie, Facultés de Médecine/Pharmacie, F-63000 Clermont-Ferrand, France; Inserm, U1107 Neuro-Dol, F-63001 Clermont-Ferrand, France

**Keywords:** conditioned, diffuse noxious inhibitory controls, fibromyalgia, milnacipran, pain modulation, randomized clinical trial

## Abstract

**Background:**

The prevalence of fibromyalgia increases worldwide and is characterized by widespread and chronic pain. Treatment is difficult and includes both drug and non-drug approaches. Milnacipran, an antidepressant, is used for fibromyalgia, with a possible beneficial effect on central pain modulation. Our hypothesis is that the efficacy of milnacipran in fibromyalgia depends on the performance of pain inhibitory controls.

**Methods/design:**

A randomized, double blind, clinical trial (NCT01747044) with two parallel groups, in 48 women with fibromyalgia, is planned in the Clinical Pharmacology Center, University Hospital, Clermont-Ferrand, France. Conditioned pain modulation (estimated with thermal stimuli using a numeric pain rating scale), the primary endpoint measure, is evaluated before and one month after treatment with milnacipran or placebo. Secondary outcome measures include the predictability of pain descending pathways performance for milnacipran efficacy, tolerance and cognitive function. Data analysis is performed using mixed models; the tests are two-sided, with a type I error set at alpha = 0.05. Not only will this trial allow estimation of the beneficial effect of milnacipran on pain and on descending pain pathways but it will also evaluate whether the performance of this modulatory system could be predictive of its efficacy in alleviating pain.

**Discussion:**

This method would allow clinicians to take a pro-active attitude by performing a rapid psychophysical test before starting milnacipran treatment and would avoid unnecessary prescription while preventing therapeutic failure in patients who often face this recurrent problem.

**Trial registration:**

ClinicalTrials.gov NCT01747044.

## Background

Fibromyalgia has a prevalence estimated at 2 to 5% of the general population in Europe [1] and in the United States of America [2] and is approximately seven times more common in women than in men [3]. Diagnostic criteria have been validated since 1990 by the American College of Rheumatology and have been enlarged in the 2010 revision to consider not only tender points but also cognitive problems and the extent of somatic symptoms [4]. The pathophysiology of fibromyalgia is uncertain but a dysfunction of descending pain inhibitory pathways [5-7] has been described as a cause or a consequence of the pathology [8]. These pathways originate in the brainstem and are involved in diffuse noxious inhibitory controls (DNICs) that are part of a central pain modulatory system relying on spinal and supraspinal mechanisms [9]. This pain modulatory system can be easily triggered and studied in experimental settings [10,11] using the conditioned pain modulation test, where the administration of two simultaneous painful stimuli typically results in pain inhibition [12,13]. Stimulation of DNICs is commonly used to evaluate any impairment of descending pain modulation.

Antidepressants have a mechanism of action that involves a number of neuromediators (serotonin, noradrenaline) within the brain-brainstem-spinal-cord axis and affects pain descending pathways [14]. Duloxetine and milnacipran are serotonin and norepinephrine reuptake inhibitors and have been approved for treatment of fibromyalgia by the Food and Drug Administration [15-17] for their analgesic properties [14]. Experiments on the safety, tolerability, and clinical benefits of long-term milnacipran use in patients with fibromyalgia have shown good results [18]. A review of four double blind studies focused on the efficacy of milnacipran in fibromyalgia [18]: a substantial improvement on global status was observed and more than 30% pain relief was obtained but only in 40% patients.

Considering this large variability in response to milnacipran, it is expected that a number of patients will have no alleviation of pain: it would be beneficial for the patient to be able to predict the efficacy of milnacipran in pain associated with fibromyalgia before the prescription of the drug. It would also be important to evaluate cognitive function, which is frequently impaired in chronic pain [19]. This information would enable prescription of the drug to be avoided if no efficacy is expected, and would avoid therapeutic failure in patients who often stop their treatment because of lack of efficacy. Such a predictive approach has been performed with duloxetine in another pathology, painful diabetic neuropathy [13], where the authors have shown that pain alleviation may be predicted from pre-treatment conditioned pain modulation. Such an approach is quite interesting in the context of milnacipran as DNICs are involved in the mechanism of action of both drugs [12,17]. In this trial, we set the mechanistic hypothesis that (1) milnacipran restores the function of descending inhibitory pathways and concomitantly decreases the pain experience in fibromyalgia patients, and (2) this efficacy could be predicted from the performance, before treatment, of pain descending pathways when activated by conditioned pain modulation.

## Methods/design

This study is a prospective, randomized, controlled, double blind clinical trial of two parallel groups, milnacipran versus placebo, in 48 fibromyalgia patients. It is carried out in the Pain Clinic and Clinical Pharmacology Center, University Hospital, Clermont-Ferrand, France. The research ethics committee (Comité de Protection des Personnes Sud-Est 6) gave its approval on October, 18, 2012 (institutional review board number 00008526/AU987), and the Agence Nationale de Sécurité du Médicament et des Produits de Santé on September, 25, 2012, and this trial is registered in ClinicalTrials.gov (trial number NCT01747044).

Patients come twice to the Pain Clinic and Clinical Pharmacology Center, for inclusion and randomization and 1 month later for reassessment.

Eligibility criteria are verified with a complete clinical exam, matching the American College of Rheumatology’s definition of fibromyalgia. Patients meeting the inclusion criteria sign a consent form after receiving oral and written information about the study. At baseline, pain is assessed by a numeric pain rating scale and patients undergo psychophysical and cognitive ability tests. Patients are then randomly assigned to milnacipran (n = 24) or placebo (*n* = 24) for one month’s treatment, with the following titration: 50 mg on the first three days, 75 mg from day 4 to day 6 and 100 mg from day 7 to 1 month. Evaluations and tests are then repeated 1 month later at visit 2. To maintain good compliance and to verify adverse events, patients are telephoned once a week by a clinical research assistant until visit 2.

### Objectives

The primary objective of this trial is to assess whether milnacipran has an influence on DNIC status, by measuring if there is a difference between the results of the conditioned pain modulation test in milnacipran and placebo groups.

The secondary objectives are to assess:

- Whether the DNIC status could be predictive of milnacipran efficacy in fibromyalgia,
- The evolution of pain threshold with milnacipran,
- The impact of milnacipran on cognitive parameters; comprehension tests, executive function, memory, attention and decision making,
- Tolerance towards milnacipran.

### Participants and setting

#### Inclusion criteria

Patients are eligible for this study if they are over 18 years old with a confirmed diagnosis of fibromyalgia, are able to understand and willing to follow the study protocol and have given informed consent.

#### Exclusion criteria

Patients will not be accepted if there is contra-indication of milnacipran treatment, if they have hypertension or heart disease, known renal impairment, a medical or surgical history incompatible with the study, or a psychiatric disorder, or if they have shown signs of suicidal behavior or significant suicidal ideas. This study also excludes patients with diabetes, patients with acute and chronic intoxication with alcohol, hypnotics, analgesics, or opioids, patients taking diuretics or a treatment inducing hyponatremia, patients taking non-steroidal anti-inflammatory drugs, oral anticoagulants, aspirin or other drugs that are likely to induce bleeding, and patients taking drugs with serotonin and noradrenaline reuptake inhibition, digitalis, cytochrome P450 1A2 inhibitors, or 5-hydroxytryptamine serotonin receptor agonist. Women of childbearing age not using an effective contraceptive will be excluded, as will pregnant or breastfeeding women, patients taking part in another interventional trial or patients unable to understand the patient information and the informed consent form.

### Eligibility and randomization

Patients are recruited during their admission to the Pain Clinic and Clinical Pharmacology Center. Before enrolment, informed consent is obtained from each patient in the presence of the investigator. Randomization will be conducted to balance group sizes according to a computer-generated allocation sequence by blocks with Stata software by a statistician independent of the protocol [20]. The randomization sequence is generated using random blocks and the size of the blocks is unknown, to avoid guessing during the trial. Subjects are then randomized to milnacipran or placebo groups. Double blinding will be fully respected with the patients and the members of staff. A research nurse who is only involved in drug allocation will be in charge of drug administration. Treatments are prepared in the Central Pharmacy of the University Hospital with double blinding according to Good Pharmaceutical Practice. The milnacipran and placebo will be administered in the form of white capsules that look alike.

### Definition of outcome measures

#### Primary outcome measure

The primary outcome measure is the change of conditioned pain modulation effect induced by milnacipran compared with placebo.

#### Secondary outcome measures

Secondary outcome measures are the assessment of the evolution of pain threshold, DNIC status, milnacipran efficacy and tolerance, and cognitive status.

The summary of the different evaluations and tests is reported in Table 1.

**Table 1:** **Summary of evaluation for a patient**

**Study visit**	**Visit 1**	**Phone call**	**Visit 2**
	**Day 0**	**Day 0 + 7 days, 14 days, 21 days (interval 1 day)**	**Day 28**
***Clinical interviews***			
Informed consent	×		
Inclusion and exclusion criteria	×		
Clinical examination: diagnosis of fibromyalgia	×		
***Measures***			
Numeric pain rating scale	×		×
Assessment of pain threshold to a thermal stimulus	×		×
Test-stimulus intensity ‘pain 60/stimulus’	×		×
Conditioned pain modulation test	×		×
Cantab® tests	×		×
***Treatment***			
Randomization: delivery of therapeutic units	×		
Reporting of adverse events and concomitant treatments	×	×	×
Treatment compliance		×	×
Reporting of adverse events and concomitant treatments	×	×	×
Return of therapeutic units			×

### Conditioned pain modulation

Conditioned pain modulation is evaluated before treatment randomization (visit 1, CPMv1) and after 1 month treatment (visit 3, CPMv3). The milnacipran and placebo differences (CPMv3 − CPMv1) are then compared. With pain intensity rated by the patient on a numeric scale from 0 (no pain) to 100 (worst pain possible), the conditioned pain modulation is obtained with the following experiment. An Advanced Thermal Stimulator thermode (Medoc Ltd, Ramat Yishai, Israel) is applied to the volar side of the patient’s dominant forearm. The Medoc PATHWAY system delivers stimulation at the predetermined pain threshold for 10 seconds and the patient rates her pain using the numeric scale. Then the Medoc PATHWAY system delivers a stimulus at the same temperature for 30 seconds and the patient rates her pain using the numerical scale. After the end of these two stimuli (15 minutes), the patient puts the non-dominant hand into a water bath at 46.5°C for 60 seconds. After having dried her hand, the patient has a similar sequence of stimuli for 10 and 30 seconds with pain evaluation using the numeric pain rating scale. The aim of this test is to trigger the stimulation of pain descending pathways. If these pathways are functional, the second series of stimuli will be less painful than the first one because of the inhibitory effect on pain stimuli. It is the reverse if these pathways are not functional, as described in a number of chronic pain situations including fibromyalgia [11,21]. The amplitude of the conditioned pain modulation is given by the difference between the pain scores on the numeric pain rating scale before and after the immersion of the non-dominant hand.

### Assessment of pain thresholds with a thermal stimulus

The Advanced Thermal Stimulator thermode (30 × 30 mm) connected to the Medoc PATHWAY system is applied to the volar side of the patient’s dominant forearm. From the baseline value of 32°C, the Medoc PATHWAY system delivers an adjustable temperature peak (in cold and heat, depending on a regular increase of 1°C increments) and is controlled by rapid feedback. This device is used to evaluate the pain threshold to heat and cold by calculating the mean of three measures. This technique was recently used successfully in neurophysiology studies in human beings [11] and showed similar results to those obtained with a laser [22].

### Test-stimulus intensity ‘pain 60/stimulus’

The Advanced Thermal Stimulator thermode is applied to the volar side of the patient’s dominant forearm. From the baseline value of 32°C, the PATHWAY system delivers a series of peaks starting at 1°C above the pain threshold. Then the temperature increases by 1°C until the patient rates the pain intensity as equivalent to 6/10 on the numeric pain rating scale.

### Cognitive tests

The Cambridge Neuropsychological Test Automated Battery (Cantab®, Cambridge, UK) [19] is a battery of 22 neuropsychological tests, administered to subjects using a touch screen computer and a press-pad. The tests selected are: Motor Screening Test, Stockings of Cambridge, Reaction Time, Cambridge Gambling Task, and Graded Naming Test.

### Data handling and record keeping

All original records, such as consent forms and case report forms, will be archived at the trial site for 15 years. The database file will be anonymized and will also be maintained for 15 years.

### Sample size calculation

According to our previous work and a study of the literature [13], we calculate that a sample size of *n* = 24 patients per randomized group would provide 90% statistical power of detecting an absolute difference of 15 points in the primary outcome, that is, the conditioned pain modulation difference between milnacipran and placebo (with a standard deviation of conditioned pain modulation variation equal to 16 points), at a two-sided alpha level of 0.05. A total of 48 patients will be included.

### Statistical analysis

Statistical analysis will be conducted on an intention-to-treat principle using Stata software, version 13 (StataCorp, College Station, TX, USA). A two-sided *P* value of less than 0.05 will be considered to indicate statistical significance. Baseline characteristics will be presented for each randomized group as the mean ± standard deviation or the median (interquartile range) according to statistical distribution for continuous data, and as the number of patients and associated percentages for categorical parameters. Comparisons between independent groups will be analyzed using the Chi squared or Fisher’s exact test for categorical variables, and by Student’s *t*-test or the Mann–Whitney test for quantitative parameters (notably primary outcome, temporal summation, test-stimulus intensity ‘pain 60/stimulus’, pain rating on the numeric pain rating scale, cognitive parameters, with normality verified by the Shapiro-Wilk test and homoscedasticity by the Fisher-Snedecor test. As suggested by Vickers and Altman [23], the primary analysis will be completed by an analysis of covariance considering conditioned pain modulation at 1 month as a dependent variable, and group and baseline value of conditioned pain modulation as independent parameters. Finally, to study whether the DNICs (assessed using the conditioned pain modulation test) in patients with fibromyalgia would be predictive of milnacipran efficacy, multivariate models such as regression analysis (milnacipran efficacy evaluated by conditioned pain modulation difference taken as a dependant variable [13]) will be performed considering the randomization group and the baseline values of conditioned pain modulation and pain as covariables. Secondarily, a sensitivity analysis concerning missing data will be considered to study their nature (not missing at randomization, missing at randomization and so on) and to propose the most appropriate imputation approach.

## Discussion

Evaluation of predictive factors of pain development and pain alleviation is very important for treatment optimization [24]. Pain alleviation in fibromyalgia is very difficult, as the response to analgesics is very variable. Milnacipran is particularly recommended for the management of fibromyalgia [16] but only 40% of patients are relieved (30% less pain) [18]. Adding to this high variability in the response to milnacipran, discontinuation of treatment is also due to adverse events in 25% of patients compared with 12% in those receiving placebo [25]. Considering that many patients have no improvement of pain symptoms, it is therefore important to explore the status of the pain modulatory system and evaluate whether DNICs could be a predictive factor of the efficacy of milnacipran in patients with fibromyalgia. The comparison of the performance of descending inhibitory pathways when triggered by a painful stimulus before and after treatment gives information on the amplitude of pain modulation and control. It may help to predict non-responders, as shown for duloxetine [13], and identify the risk of developing chronic post-operative pain [26]. This study aims first to evaluate the state of functioning of the DNICs in patients with fibromyalgia and then to estimate if it might be a predictive factor of pain alleviation. If such an impact is demonstrated, clinicians could perform a simple psychophysical test to test the DNIC performance before prescribing milnacipran. Considering the tight links between chronic pain and cognitive impairment [19], the study will also assess the impact of milnacipran on several cognition domains. This approach could help to optimize treatment, would avoid useless prescription, prevent unnecessary adverse events, and, more generally, would prevent recurrent therapeutic failure in non-responder patients.

## Trial status

Recruitment started in April 2013. At the time of writing, 26 patients had been recruited.

## Abbreviations

CPMv1: conditioned pain modulation test, visit 1
CPMv3: conditioned pain modulation test, visit 3
DNIC: diffuse noxious inhibitory control
